# Long-Distance Wind-Dispersal of Spores in a Fungal Plant Pathogen: Estimation of Anisotropic Dispersal Kernels from an Extensive Field Experiment

**DOI:** 10.1371/journal.pone.0103225

**Published:** 2014-08-12

**Authors:** Adrien Rieux, Samuel Soubeyrand, François Bonnot, Etienne K. Klein, Josue E. Ngando, Andreas Mehl, Virginie Ravigne, Jean Carlier, Luc de Lapeyre de Bellaire

**Affiliations:** 1 CIRAD, UMR BGPI, Montpellier, France; 2 INRA, UR546 Biostatistics and Spatial Processes (BioSP), Avignon, France; 3 Centre Africain de Recherches sur Bananiers et Plantains (CARBAP) – Njombe, Cameroon; 4 Bayer CropScience AG, Monheim, Germany; 5 CIRAD, UPR Systèmes de culture bananiers plantains ananas, Montpellier, France; University of Nebraska-Lincoln, United States of America

## Abstract

Given its biological significance, determining the dispersal kernel (i.e., the distribution of dispersal distances) of spore-producing pathogens is essential. Here, we report two field experiments designed to measure disease gradients caused by sexually- and asexually-produced spores of the wind-dispersed banana plant fungus *Mycosphaerella fijiensis*. Gradients were measured during a single generation and over 272 traps installed up to 1000 m along eight directions radiating from a traceable source of inoculum composed of fungicide-resistant strains. We adjusted several kernels differing in the shape of their tail and tested for two types of anisotropy. Contrasting dispersal kernels were observed between the two types of spores. For sexual spores (ascospores), we characterized both a steep gradient in the first few metres in all directions and rare long-distance dispersal (LDD) events up to 1000 m from the source in two directions. A heavy-tailed kernel best fitted the disease gradient. Although ascospores distributed evenly in all directions, average dispersal distance was greater in two different directions without obvious correlation with wind patterns. For asexual spores (conidia), few dispersal events occurred outside of the source plot. A gradient up to 12.5 m from the source was observed in one direction only. Accordingly, a thin-tailed kernel best fitted the disease gradient, and anisotropy in both density and distance was correlated with averaged daily wind gust. We discuss the validity of our results as well as their implications in terms of disease diffusion and management strategy.

## Introduction

The dispersal kernel, *i.e.*, the probability density function of dispersal distances relatively to a point source, constitutes the most basic and synthetic descriptor of the dispersal process [1]. Interests in dispersal kernels increased recently, jointly with the recognition of the importance of long distance dispersal (LDD). The magnitude and frequency of LDD events, determining the shape of the tail of the dispersal kernel, has been shown to play an important role in a variety of ecological and genetic processes such as *i)* the rate of spread of an expanding population [2], [3], *ii)* the spatial distribution of neutral genetic diversity [4]–[8], *iii)* the transfer of genes between locally adapted populations [9] and *iv)* the response to climate changes [10]. Dispersal kernels have thus been estimated in various taxonomic groups dispersing actively (insects, [11], [12]; birds, [13]; mammals, [14]), or passively (plants, [15]–[17]; larvae of marine organisms, [18]–[20]). For self-dispersing pathogens, *i.e.*, those that do not require any host or biological vector movement to disseminate such as most spore producing fungal plant pathogens, a better knowledge of wind-dispersal processes, including LDD is required to better predict patterns of disease spread at both local and global scale [21]–[23] and/or to define new efficient control strategies [24]–[27]. However, despite its importance, LDD remains challenging to characterize accurately [28] and very little is known about the dispersal ecology of self-dispersing pathogens.

Three complementary approaches are used to study wind-dispersed propagules such as pollen, seeds or spores [29]. The first relies on mechanistic models that include a fine modelling of physical processes contributing to the release, transport and deposition of propagules [30]–[33]. These approaches have much developed in the last years, especially thanks to the new possibilities to conduct intensive numerical simulations that demonstrate the effect of air turbulence on LDD (see [34] for a review on seeds). The second approach estimates gene flow from genetic data through measures of genetic differentiation among populations or individuals (i.e., indirect approaches) [35]. Indirect methods generally allow the estimation of a single synthetic parameter (e.g., average dispersal distance per generation) that quantifies gene flow averaged over time and space. They cannot differentiate different types of dispersal events, especially LDD (but see attempts in [36]–[39]. The third approach is based on direct measurements of dispersal distances, achieved by tracking propagule movement (see [40] for a review on such direct methods in plants), often using genetic markers. While direct approaches are costly, fastidious and time-consuming [41], they allow an experimental measure of the magnitude and frequency of LDD events through the estimation of the dispersal kernel [1].

In principle, dispersal kernels can experimentally be estimated from the real-time tracking of propagules (Lagrangian methods) or from the amount and/or diversity of propagules observed at different distances from a source (Eulerian approach) [40]. Lagrangian methods have mostly been applied to animals or animal-dispersed propagules (e.g., seeds, pollen) using mark/recapture or tracking designs [1]. They can barely be applied to wind-dispersed pathogen propagules because of their small size (but see [42]). In such situation, the Eulerian approach appears to be more suited in determining how many propagules originating from an isolated or identifiable source of inoculum arrive at specific distant location. While some experiments based on the Eulerian approach have successfully been performed on plants (e.g., [40], [43]–[47]), their application to wind-borne plant pathogens have been so far extremely limited to small-scale plots and/or artificial (e.g., wind tunnel) conditions [48]–[52]. This may be because Eulerian approaches require a number of precautions in the experimental design [40], which can be fastidious when dealing with disease gradients caused by infectious spores. First, external contamination must be avoided, which requires performing the experiment in a disease-free area or to use a traceable source of inoculum. Second, secondary dispersal events (which occur when inoculum disperse from a plot that has itself been infected by the primary source of inoculum) must be either distinguishable or avoided. Third, the sizes of the experimental site and of the inoculum source are both critical and must be large enough to provide measurable disease levels at long distances. Fourth, because spore dispersal might be anisotropic (i.e., direction-dependent) [48], trapping should be arranged in several directions [53]–[55]. Fifth, trapping design as well as the sizes and shapes of trap plots need to be carefully conceived [40], [56], which generally requires *a priori* knowledge about the dispersal abilities of the species under study [1].

In this article, we show how taking such precautions in the design of a large-scale trap experiment, combined with dedicated statistical analysis, allow estimating anisotropic dispersal kernels under natural conditions in a wind-dispersed plant pathogen. We focus on the fungus *Mycosphaerella fijiensis*, the causal agent of Black Leaf Streak Disease (BLSD) of banana and plantain. This ascomycete species, considered as one of the most important crop pathogen in the world [57], is a major cause of yield loss and the massive use of fungicides for its control is detrimental to the environment [58], [59]. In addition to accidental transport of infected plant material, the species naturally spreads via the dispersal of both sexually-produced (ascospores) and asexually-produced (conidia) spores. Lesions being asynchronous, both types of spores are continuously produced: conidia are produced first in the younger stages while ascospores are produced in necrotic spots at the latest stage of the disease. Air release of both types of spores is noticeably different and influenced by climatic conditions [60]. Conidia are lightly attached to conidiophores, laid on the leaf surface, and are blown off passively by wind or water, whereas ascospores are expulsed actively into the air from perithecia during wet periods [61], [62]. Previous studies used spore traps to clarify the relative importance of ascospores *vs.* conidia dispersal inside and outside plantations [61]–[64]. It was concluded that ascospores might be carried over long distances whereas conidia would be dispersed mainly over short distances or at the plant level (see review in [65]). Other studies were carried out to measure dispersal distances through spore trapping or disease gradient analysis but none estimated accurately dispersal kernels: either external contaminations were impossible to avoid ([65], Abadie et al., *unpublished data*) or disease gradients were observed over several disease cycles [62], [66]. Interestingly, Rieux et al. [67] recently generated the first indirect (based on genetic data) estimate of dispersal in *M. fijiensis*, which makes this pathogen species a good model to directly assess the dispersal kernel as comparison between direct and indirect estimations can prove useful [68].

## Materials and Methods

### Experimental design

Field work was performed on a private piece of land, belonging to the SOCAPALM Company, who provided us the permit to perform our experiment on their domain. Our field study did not involve endangered or protected species Two experiments were conducted within an industrial rubber tree plantation in southwest Cameroon (4°14′N, 9°57′E) in a flat area with no obstacles to spore dispersal (rubber trees were young and thin). Banana trap plants (cultivar Grande Naine) were planted in June 2010 following two trapping designs and controlled infected plants were introduced in the centres of the two designs on October the 20th (day 0, D_0_).

#### Ascospore experimental site

**Figure 1:**
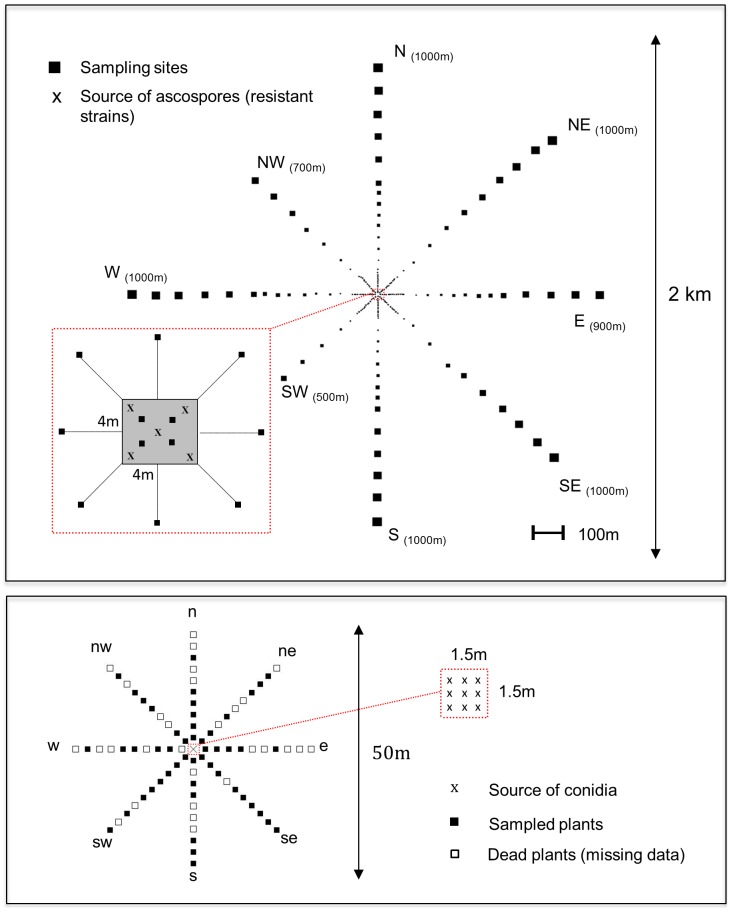
Plots layout of both ascospores and conidia experimental designs implanted in Cameroon. For the ascospores system (upper panel), the sizes of the squares (schematizing sampling sites) are proportional to the number of trap plants and the number into brackets indicates for each axis the distance between the centre and the most distant sampling site.

Trap plants were planted at 27 different distances, from 4 to 1000 m, on each of eight transects (E, NE, N, NW, W, SW, S, SE) radiating from a 4×4 m central square (Figure 1). In order to measure accurately short-distance gradient, single banana trees were densely planted (one plot every 8 meters) up to 104 m from the centre. At larger distances (from 104 to 1000 m), the distance between consecutive plots was increased (one plot every 50 meters) and the number of trap plants per plot was doubled every 100 m (i.e, 1 plant at 100 m, 2 plants at 200 m and 10 plants at 1000 m, see Appendix S1 in File S1) to improve rare long distances dispersal events detection, as previously shown using Monte Carlo simulations [56]. In directions E, NW and SW, the dimensions of the rubber tree plot did not allow the installation of plants at the largest distances. A total of 511 banana plants were planted in 192 plots. We have chosen the distances at which sampling was denser or looser following previous empirical knowledge about *M. fijiensis* ascospore dispersal (Abadie et al., *unpublished data*). The total number of potted plants was fixed by practical experimental constrains.

#### Conidia experimental site

Traps were planted in an open area without rubber trees, 3 km SW away from the ascospore site. Because we expected that conidia disperse much closer than ascospores, the size of this experiment site was smaller. One banana trap plant was planted every 2.5 m from a central point up to a distance of 25 m in each of eight directions (indexed e, ne, n, nw, w, sw, s, se) (Figure 1).

### Initial conditions

Two distinct sources of inoculum, highly resistant to Quinone outside inhibitors fungicides (QoI) were carefully prepared for ascospores and conidia (See Appendix S2 in File S1). The number of spores potentially released by each of the two inoculum sources was both experimentally and theoretically assessed (See Appendix S3 in File S1). During the five months prior to the start of the experiment (D_0_), standard cultural practices including fertilization were applied. The week before D_0_, all banana trap plants were sprayed with QoI fungicide (under conditions described in Appendix S2 in File S1) and all their leaves were removed except the last unfurled leaf (called the cigar), and the first leaf under the cigar (F1). This aimed at eliminating all undesirable inoculum sources inside the experimental site. At D_0_, most banana plants showed good physiological conditions in the ascospore site, whereas 29 plants over 80 (36%) died due to bad soil conditions in the conidia site. The inoculum sources were then installed at the centre of each of the two experimental sites (Figure 1 & Figure S2-2 in File S1). Fungicide treatments were applied weekly until the end of the experiments.

### Measuring disease gradient

Observations were realized on the leaf that was unfurled at D_0_. Inoculum source was removed once we detected the first symptoms. The first lesion (i.e., characteristic streak typic of *M. fijiensis* infection [69]) was observed 17 days after inoculation on both experimental sites. This is considered as the latent period (*LP*). To avoid assessing lesions from a second generation of spores the final counting of lesions was made between days 26 and 30. Only stage 2 and 3 lesions (i.e., streaks longer than 1 mm) were considered since stage 1 lesions can be confused with other leaf injuries [69]. For each trapping plot, we noted TNL, the total number of lesions and DL- the density of lesions (in number of lesions/leaf m^2^). The area of each leaf analysed was estimated from leaves length and width as *length*× *width* × 0.83 [70].

### Meteorological data

We used a WS2800 (La crosse Technology) weather station to record wind gust and speed at a frequency of one measurement every 15 minutes. Daily rainfall was recorded using a rain gauge. We computed two different indices reflecting wind patterns. First we calculated the cumulative wind speed in each direction as the sum of wind speeds blowing in that direction over all measurement points. Second we considered the frequency of wind records in each specific direction. We also computed those indices considering only the measures between 5 and 9 am because intense ascospore discharge occurs during this period in *M. fijiensis* due to rapid variations in relative humidity induced by dew or higher rainfall [61].

### Molecular detection of QoI-resistant strains

Molecular detection of QoI resistance was performed at three instances. We first evaluated resistance in the source of inoculum. To do so, we collected 30 lesions/plant over 10 plants at 7 different dates during the preparation of inoculum. Secondly, we checked the absence of resistance in the experimental environment before inoculation. For this purpose, we collected 30 lesions/plant over one plant at the extremity of each axis, one plant in the centre of the experimental site, and 15 plants outside the experimental site. Thirdly, we evaluated the rate of non-resistant lesions at D_30_ by constantly and randomly collecting up to 32 lesions per plot after lesion counting. For plots containing several plants, lesions have been randomly sampled among the different plants. Bulks of maximum 10 lesions from the same leaf were constituted for DNA extraction and the frequency of the G143A mutation conferring resistance to QoI fungicides in *M. fijiensis* was assessed for each bulk through pyrosequencing (See Appendix S4 in File S1). We denote GNL, the number of genotyped lesions, and GNRL, the number of genotyped resistant lesions.

### Statistical analyses

We assumed that the total number of resistant lesions (TNRL) on a leaf follows either a Poisson or a negative-binomial distribution with mean *S*(*x*, *y*), the infectious potential at location (*x*, *y*). We further assumed that GNRL follows a hypergeometric distribution with parameters TNL, TNRL and GNL, because sampling is made without replacement [71]. Details and equations for the likelihood calculation of the dispersal models are given in Appendix S5 (in File S1).

#### Anisotropic dispersal kernels

Dispersal is anisotropic when propagules disperse differently depending on the direction. We focused on two types of angular dependence, anisotropy in density and distance, represented by two independent angular functions [54], [55]. The first (*f*(*φ*))describes the distribution of spore dispersal directions. The second (*g*(*φ*)) () provides the expected distance travelled, given the direction. We used the unimodal von Mises functions [72] (*i.e.*, with only one main direction) for those two angular functions (See Appendix S6 in File S1 for details).

The infectious potential, *S*(*x,y*), was computed as:
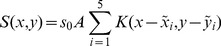
(1)where *s_0_* represents the number of spores released from each of the 5 sources (hereafter called source intensity) assuming that the 5 sources produced the same amount of inoculum, *A* represents the surface area of a trap leaf, 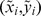 is the location of source *i*, and *K*(*x*, *y*) is a 2D anisotropic kernel.

We considered 4 different anisotropic kernels: exponential [36], exponential power (thin or fat tailed according to the value of the shape parameter [36], geometric (fat tailed, [36]) and WALD (fat tailed, a closed-form simplification of a mechanistic model for seed dispersal by wind [73]).
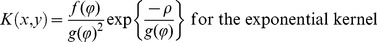
(2)

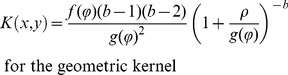
(3)

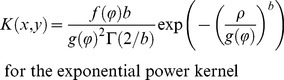
(4)

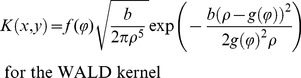
(5)where 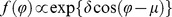 and 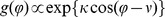 are Von Mises density functions (See Appendix S6 in File S1 for details on parameters and normalizing constants). In equations 2–5, (*ρ*, *φ*) holds for the polar coordinates of (*x*, *y*), *b* for the shape parameter and Γ for the gamma function.

#### Parameter estimation and tests of significance

In the different models, 6 or 7 parameters were estimated: the source intensity *s_0_*, the density (*μ* and *δ*) and the distance (*v* and *κ*) direction parameters of the von Mises functions, and *g*_0_, a parameter proportional to the average distance travelled. For geometric, exponential power and WALD kernels, parameter *b* additionally describes the shape of the tail. When considering the negative-binomial distribution, we jointly estimated the associated dispersion parameter *τ* (See Appendix S5 in File S1 for details). All parameters were estimated simultaneously by maximizing the likelihood using an adaptive barrier algorithm [74]. The AIC criterion was used to investigate whether the directional density function is uniform (*δ* = 0) or not (*δ*>0), the mean distance travelled is constant over all directions (*k* = 0) or not (*k*>0) and for model selection. The 95% confidence intervals for parameters were computed for the best model using 1000 nonparametric bootstrap [75]. All calculations were realized using the R software [76]. The code used to perform statistical analyses is given in File S2.

#### Mean distance travelled by spores and standard deviation of parent-offspring dispersal distances (σ)

We computed different types of mean distance travelled by spores. The first one was calculated from the raw data (i.e., without fixing any kernel), and on each direction independently as
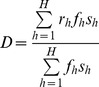
(6)To obtain independent values for all 8 directions, we divided the total surface of the experimental area *S* into 8 circular sectors, each divided into H sectors of circular ring including one sampling plot, the *h* ^th^ sector of circular ring being of surface *s*_h_, of density in resistant lesions *f*_h_, at distance *r*_h_ from the centre The second estimate was obtained from the inferred dispersal kernel (i.e., averaged over the different directions by taking the anisotropy patterns into account) with or without considering the tail of the kernel at distances longer than 1 Km, as detailed in Appendix S6 in File S1.

From the estimated kernels we also computed *σ*, the standard deviation of parent–offspring axial dispersal distances (also known as the average quadratic axial travelled distance). We computed one value for each of the 8 directions radiating from the inoculum source for ascospores only without considering the tail of the kernel at distances longer than 1 Km (Appendix S8 in File S1).

#### Correlation with wind patterns

We used the Spearman rank correlation coefficient and the associated two-sided *p*-value to test for significant correlation between spore density or distance travelled by spores and wind patterns.

## Results

### Lesions counting

#### Ascospores

**Figure 2:**
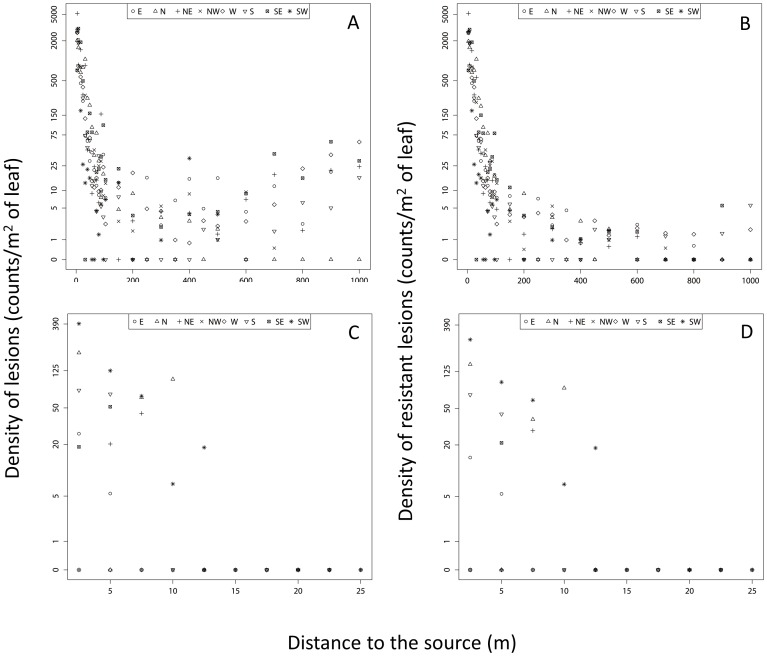
Disease gradient measured in the field. Density of lesions (i.e., DL, A & C) and density of resistant lesions (i.e., DRL, B & D) as a function of geographical distances from the central source. Top: ascospores site (A & B); Bottom: conidia site (C & D). The different axis of the experimental design are represented separately using different symbols. DL and DRL are plotted in “log (1+the value)” scale but values are indicated in natural scales.

Lesions were detected in 168 of the 192 trapping plots (87.5%). The density of lesions (DL) observed on each plot ranged between 0 (24 plots) and 5182 lesions.m^−2^ (NE_4_, the site localized 4 m North-East direction away from the source). A sharp decrease in the disease gradient was observed over the first 100 meters in all directions (see Figure 2A). In directions W, S, NE and SE, the DL increased at greater distances from the source (between 800 and 1000 m), suggesting external contamination from the vicinity of the experimental site in these directions.

#### Conidia

We observed lesions on 16 plants (31%). The DL ranged between 0 (35 plots) and 392 (SW_2.5_). No lesion was detected at a distance larger than 12.5 m from the central source (Figure 2C).

### Molecular detection of resistance

High levels of resistance (96 and 98% for conidia and ascospore respectively) were monitored in the two distinct sources of inoculum (See Appendix S2 in File S1). Before inoculation, none of the 720 lesions or necrotic tissue sampled inside or outside the experimental site displayed the QoI-resistance SNP. After lesion counting on the ascospore experimental site, the percentage of resistance detected varied from 0% (mainly for plots situated at extreme distances, close to the borders of the experimental site) to 100%, with an average value of 75%. We observed resistant lesions up to 1000 m from the source (DRL = 1.8 lesions.m^−2^ at W_1000_ and DRL = 5.6 lesions.m^−2^ at S_1000_) suggesting the occurrence of LDD events (Figure 2B & Figure S7 in File S1). On the conidia experimental site, the percentage of resistance detected varied from 0% (at two plots) to 100%, with an average value of 71%. However, unlike the situation at the ascospore site, the frequency of susceptible strains was not higher at the border of the experimental design (Figure 2 C&D).

### Weather report

The highest cumulative wind speed and frequency over the dispersal period was in the NE to SW direction (See Figure S9 in File S1). However, for data recorded between 5 and 9 a.m the highest cumulative wind speed were in the SW to NE and S to N directions. Rainfall occurred on 6 days over this period and represented a total of 109 mm.

### Statistical analysis

On both the ascospores and conidia datasets, the negative-binomial distribution was preferred to the Poisson distribution for TNRL as the former led to smaller AIC values (See Table S5.1 in File S1). As reported in Table 1, the dispersion parameter in the negative-binomial distribution was smaller (i.e, higher value of *τ*) when assuming the exponential power kernel.

**Table 1:** Ascospores and Conidia experimental sites model fit results.

Ascospores	Exponential	Geometric	WALD	Power Exponential	
N	*7*	*8*	*8*	*8*	
LL	−677.96	−589.69	−612.20	−574.22	
*S_0_*	8.50E+06	1.36E+07	1.04E+07	2.34E+07	CI (2.02E+07; 4.05E+07)
*μ*	323.72	321.43	2500.95	178.76	CI (158.13; 241.21)
*δ*	0.22	0.12	0.7	0.49	CI (0.11; 0.92)
*g_0_*	6.73E+02	2.37E+01	9.05E+52	9.32E-20	CI (1.14E-33; 2.31E-10)
*v*	258.97	233.76	246.37	**226.32**	CI (213.14; 244.65)
*k*	1.54	1.91	2.08	**3.38**	CI (2.84; 4.99)
*b*		2.002	17.329	0.064	CI (0.033; 0.077)
*τ*	0.34	1.03	0.85	1.20	
R^2^	0.21	0.43	0.41	0.55	
AIC	1369.93	1195.38	1240.41	**1164.45**	

Conidia	Exponential	Geometric	WALD	Power Exponential	
N	*7*	*8*	*8*	*8*	
LL	−62.68	−63.05	−65.58566	−59.03	
*S_0_*	7.43E+03	4.38E+06	4.71E+05	1.96E+04	CI (2.09E+03; 1.05E+05)
*μ*	227.67	224.33	215.16	**217.85**	CI (174.75; 287.05)
*δ*	2.28	3.43	4.03	**4.73**	CI (3.61; 5.09)
*g_0_*	3.23	2.01	8.64	8.08	CI (6.44; 10.37)
*v*	212.56	247.51	229.75	**225.74**	CI (194.23; 258.40)
*k*	1.18	223.4	5.03	**9.72**	CI (6.73; 12.14)
*b*		2.02	4.78	1.86	CI (1.36; 2.38)
*τ*	0.21	0.09	0.11	0.44	
R^2^	0.42	0.30	0.28	0.48	
AIC	139.36	142.11	147.17	**134.07**	

Maximum likelihood estimation of the different parameters considering the different dispersal kernels tested. N holds for the number or parameters, LL for maximum likelihood, *So* is the source strength, *μ* and *v* (degree) are the direction of the density and distance anisotropy functions respectively, *δ* and *k* are the variability around the mean for the density and distance anisotropy functions respectively, *g_0_* is a constant that cannot be compared between different kernel, *b* is the parameter of the shape of the tail and *τ* is the negative-binomial dispersion parameter. R^2^ is the coefficient of determination between observed and predicted densities of resistant lesions. AIC was calculated as AIC = 2*N - 2LL. CI for 95% confidence intervals calculated for the best model only. Best model (*i.e.*, lower AIC) and significant values of anisotropy functions are highlighted in bold.

#### Ascospores

**Figure 3:**
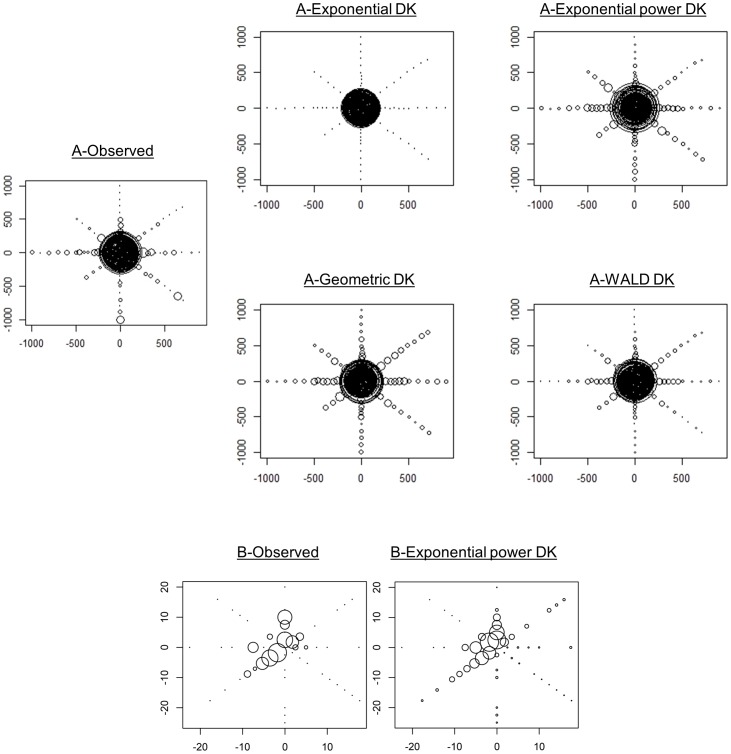
Observed *vs* predicted disease gradients. (A) Ascospore and (B) conidia experimental sites. Dispersal kernels (DK) parameter values are given in Table 1. Density of resistant lesions (DRL) is expressed in a “log (1+ of the value)” scale. Smallest dots represents sites where DRL = 0 and gaps (B) represents missing data due to plants mortality.

**Figure 4:**
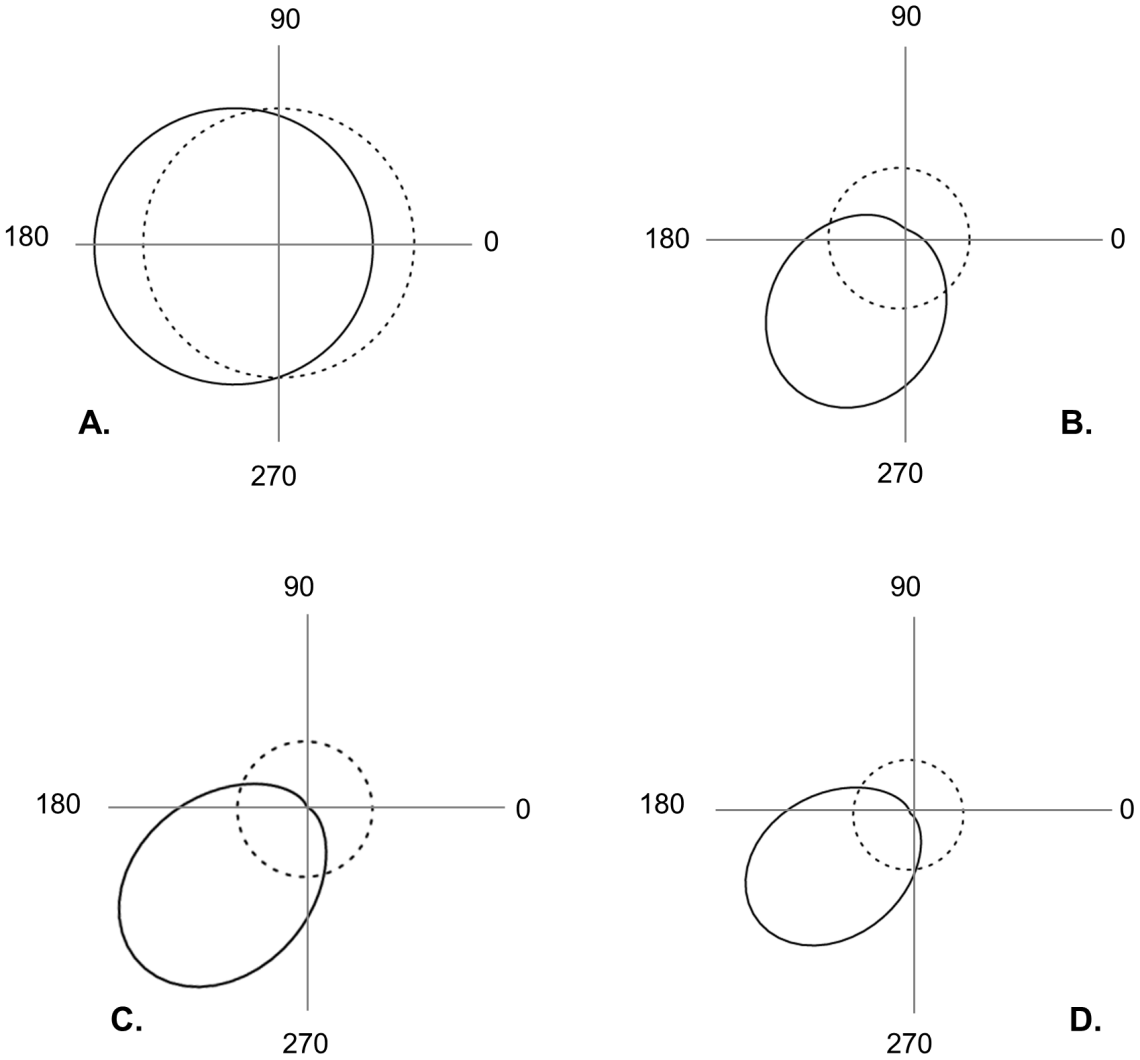
Anisotropy functions estimated from the exponential power dispersal kernel. Estimated directional functions (solid lines, A&C for the density and B&D for the mean distance) are compared to their equivalent uniform functions (dotted lines) for both the ascospores (A&B) and conidia (C&D) experimental site. See Table 1 for details on values and statistical support. Directional angles are given in degrees.

The mean distance travelled by ascospores (raw data estimate) varied across the different directions, ranging from 104 m (North direction) to 613 m (South direction) (Table 2). The exponential power kernel best fitted the data (Table 1, Figure 3 & Figure S7 in File S1). The estimated shape parameter (*b* = 0.064, CI [0.033–0.077]) indicated a fat-tailed kernel (*b*<1). The directional density and mean distance functions (calculated using the exponential power kernel) revealed contrasted patterns of anisotropy in the dispersion of *M. fijiensis* (Table 1, Figure 4). Anisotropy in density was not significant (*i.e.*, *δ* not statistically different from 0, ΔAIC = 1.03), whereas anisotropy in the mean distance travelled was significant (*i.e.*, *k* statistically different from 0, ΔAIC = 11.52). This indicated that although there is no significant preferential direction taken by spores, they are predicted to disperse further in the SW direction (angle *v* = 226 degrees). The mean dispersal distance (averaged over the various directions) obtained from the dispersal kernel shown substantial variation whether we considered or not the tail of the kernel at distances longer than the study plot maximum size (1000 m). We obtained a value of *D_1000_ = *213.83 m CI [144.23–542.17] and D*_Inf_* = 14 721.49 m CI [2 134.65–184 267.03]. Finally, the average value of *σ* computed over the 8 directions obtained by truncating the kernel over distances >1000 m was *σ* = 200.961 m/generation^1/2^ (Table S.8.1 in File S1).

**Table 2:** Mean and longest distance travelled along each direction.

Axis	Mean distance travelled (m)	Length of the axis (m)	Longest distance travelled (m)
E	273.129	900	800
N	**104.583**	1000	500
W	443.466	1000	1000
S	**613.684**	1000	1000
SE	367.537	1000	900
NE	133.473	1000	600
NW	140.638	700	700
SW	195.115	500	500

Axis	Mean distance travelled (m)	Length of the axis (m)	Longest distance travelled (m)
e	2.566	25	5
n	5.797	25	10
w	3.4	25	7.5
s	**0**	25	0
se	**0**	25	0
ne	2.6	25	5
nw	3	25	5
sw	**7.647**	25	12.5

Mean and longest distance travelled along each of the axis of both ascospores (top panel) and conidia (bottom panel) experimental site. Mean distances were obtained from raw data (Eqn. 6). Minimum and maximum values are highlighted in bold.

#### Conidia

The mean distance travelled by conidia (raw data estimate) varied between 0 m (S and SE directions) and 7.6 m (SW direction) (Table 2). The exponential power kernel provided also the best fit (Table 1) but indicated a thin-tailed kernel (*b* = 1.86, CI [1.36–2.38]). Anisotropies in both density and distance were significant (i.e., *δ* and *k* statistically different from 0, ΔAIC = 6.73 and 7.81 respectively) with more dispersal and longer dispersal distances towards the SW (angle *μ* = 215 degrees and angle *v* = 226 degrees; Table 1, Figures 3&4). In contrast with the ascopore analyse, the mean dispersal distance (averaged over the various directions) obtained from the dispersal kernel was pretty similar whether we considered the tail of the kernel at distances longer than the study plot maximum size (D*_25_* = 3.15 m CI [1.01–6.78]) or not (D*_Inf_* = 6.12 m CI [2.79–8.16]).

#### Correlation between density, distance and wind patterns

Correlation between spore dispersal (in terms of both density and distance) and wind patterns in each direction (in frequency or speed; for all records or records between 5 and 9 a.m.) provided contrasting results for ascospores and conidia. No significant correlation was found for ascospores but we found a positive correlation for conidia between wind frequency in a given direction and dispersal density (R = 0.91; P = 0.0001) or mean distance travelled (R = 0.95; P = 0.00001) (See Table S10 in File S1).

## Discussion

We provide the first direct estimate of dispersal kernel in the fungal banana pathogen *M. fijiensis*. It is also among the first estimates obtained at landscape scale, demonstrating the occurrence of LDD events in fungal plant pathogens, a group that comprises most emerging infectious plant diseases [77], and for which ecology is strikingly poorly known.

### An accurate experimental design

Our experimental design differs from the majority of previous studies focusing on self-dispersing pathogens in three points, which might have been instrumental in the success of the dispersal kernel estimation.

First, we minimized the influence of external and secondary foci contamination by *i)* inoculating a marked fungicide-resistant strain in an environment where it was absent and where the presence of the fungal pathogen was scarce and *ii)* measuring the disease gradient during a single generation. Our *a posteriori* molecular assessment results indicated that despite a weekly application of fungicide treatments, sensitive spores induced lesions on many trap plants. On the ascospore site, those sensitive strains were mostly found at the edge of the field, thus suggesting an influence of surrounding banana plantains. On the conidia experimental site, the frequency of susceptible strains was not higher at the border of the field suggesting that most of those strains could originate from the central source itself, which was composed of 96% resistant lesions according to molecular analyses. Susceptible conidia might be over-represented if the sporulation of resistant lesions was affected by a cost of QoI-resistance. Besides, recent observations showed a large variance of sporulation among young lesions from a same banana leaf (from 10 to 1000, unpublished data), which could account for a stochastic distribution of susceptible lesions within the small number of lesions counted (255 in total). Since the experiment was not designed to quantify external contamination, no further attempt was made to analyse the spatial distribution of susceptible strains or to trace back their origin.

Second, we managed to concentrate a heavy inoculum source on a tiny area and adopted a large and intensive sampling design particularly adapted to the detection of LDD events and anisotropy patterns. Those precautions were particularly efficient on the ascospore experimental site where 75% of the trap plants have been infected by a resistant spore, including two plots located at 1000 m from the source. Two previous studies managed to measure large scale single-generation disease gradients from an identified source of inoculum in a windborne pathogen fungus. First, Sackett and Mundt [78] investigated spore dispersal up to 170 m in one-dimension from a primary source of a specific strain of *Puccinia striiformis*, causing wheat stripe rust. They detected rare LDD events at 80 m from the source and showed that a non-exponentially bounded (i.e., fat-tailed) model best fitted their field data. However, they could not investigate anisotropy in their 1D design. Later, Soubeyrand et al. [54], focused specifically on the detection of anisotropic patterns for the same pathogen. They also used a specific strain of *P. striiformis* as inoculum source and assessed the disease gradient up to 400 m through a large array of small plots arranged randomly in two-dimensions around the source. They detected rare LDD events at 225 m and investigated spore dispersal anisotropy in both density and distance. However, they did not characterize the shape of the kernel tail since they fitted a unique exponential model to their data. Here, we extended this approach to three other kernel families and simultaneously tested the patterns of anisotropy in density or distance in BLSD disease gradients.

Third, we simultaneously measured disease gradients caused by both sexually- and asexually-produced spores under similar environmental conditions which, as far as we know, has never been done before. This provides a unique opportunity to compare the dispersal abilities of these two types of propagules.

### Spores dispersal in *M.fijiensis*

For ascospores we detected both a steep gradient in the first few metres of each direction and LDD events up to 1000 m from the source in two of the eight directions. Such rapid and sharp gradients have been described previously for airborne fungi [48], [78] including *M. fijiensis* [63]. However, the detection of dispersal events up to 1 km from a localized primary source of inoculum far exceeds the distance over which LDD had been characterized experimentally for any plant pathogen fungi [54], [78], even if intercontinental aerial dispersal has been suspected for some species and particularly for rusts [79]. Previous studies suggested that ascospores of *M. fijiensis*, could disperse over several kilometres [65], [66], but the occurrence of contamination (either external or due to several foci) could not be excluded. Here, our results clearly show that ascospores of this pathogen can effectively disperse at least up to 1 km in a single generation.

Following the recent developments in movement ecology [1], we used our data to estimate a 2D dispersal kernel including a full accounting of anisotropy [54], [80]. Disease gradient was best fitted by a fat-tailed exponential power. This result is consistent with theoretical considerations showing that wind dispersal is a major mechanism of LDD, and that windborne fungi frequently display such patterns [7], [48], [81]. It is noteworthy that the relation between the log of the total density of resistant lesions and distance appeared linear up to 150 m (see Figure 2b), which would be characteristic of an exponential decrease at this scale. In other words, our conclusions would have been different if we had carried out this experiment up to 150 m only, illustrating how the reliability of dispersal models is relative when field data are measured along a truncated part of the dispersal distance distribution [78], [82]. Anisotropy functions indicated no significant preferred direction for ascospores, although they dispersed on average further in the SW direction. However, because the von Mises functions we used are unimodal they can characterize only one unique preferred direction. The SW anisotropy in distance estimated could thus result from higher mean distances travelled in both the South and West directions, as suggested by the mean dispersal distances independently calculated along the 8 directions (Table 2). And the higher distances travelled in the South and West directions are probably due to the highest wind speeds recorded in those directions. Soubeyrand et al. [54] also reported two significant anisotropies that did not coincide (different directions). These authors hypothesized that several factors, such as topography, wind speed or wind direction could be implicated in this difference but the accuracy of meteorological data did not allow a more thorough understanding. Here, we hypothesize that the dispersal direction is fixed by the direction of the wind at expulsion time. In *M. fijiensis*, it has been suggested that ascospores discharge would be more important during the time interval of 5–9 a.m [61]. According to the recorded wind patterns, if most ascospores had been discharged during this period, anisotropy in density would probably have been observed in the North and North-East directions. Then, it is more probable that ascospore discharge occurred at different periods of the day with different wind directions but unfortunately the daily rainfall data do not allow a better understanding.

For conidia, the spatial patterns of resistant lesions showed a gradient in 1 of the 8 axes only (SW direction), and only up to 12.5 m. In other directions, either no lesions were detected or lesions were found only on the first plants surrounding the source. Previous data on the dispersal of *M. fijiensis* conidia were based on air trapping methods within the banana canopy (Rutter and Burt 1998) and cannot be compared to ours. However, our results are consistent with several studies on different species with comparable conidia, which found dispersion over just a few metres from the source [49]–[52]. Interestingly, disease gradients were also best fitted by an exponential power kernel but contrary to ascospores, the shape parameter indicated a thin tailed dispersal kernel. Anisotropy in density and in distance were oriented in the same direction as the strongest winds, suggesting that higher wind speeds in a given direction result in higher numbers of conidia dislodged and higher distances travelled. Such relationship between wind speeds and conidia liberation has been previously reported for several fungal pathogens [49].

The differences between ascospores and conidia in average dispersal distance, weight of the kernel tail and anisotropy patterns are likely due to contrasted physical properties and liberation mechanisms. Conidia are long, generally 5–7 septate (30–130 µm), and formed at the apex of conidiophores, directly at the leaf surface where they will be dislodged by wind. Ascospores are smaller, bi-cellular (15 µm) and formed in perithecia before being expulsed in the air after water immersion [60]. Such physical differences may lead to differences in the way air turbulences affect the two types of spores. Another non-exclusive explanation would be that both types of spores are not dispersed at the same period of the day as reported previously [61]. Recent studies have demonstrated the importance of the conditions at release on the dispersal pattern eventually realized [83]–[86]. Further developments in this way will surely improve our understanding of the *M. fijiensis* system.

### Model extrapolation and experiment limitations

We observed considerable variation between the ascopores average dispersal distance (as well as the width of its associated confidence interval) predicted by the model whether we included or not in the calculation the tail of the kernel at distances longer than 1 km (the maximum detectable dispersal distance in our study plot). As previously described [78], [82], [87], [88], this result illustrates how risky extrapolating dispersal over observed distances is, especially in the case of heavy-tailed kernel. The fact that the maximum detected dispersal distances (1 km) match the plot size indicates that ascospores dispersal in *M. fijiensis* is likely to occur at a larger scale. This observation naturally suggests that performing such a release/capture experiment over longer distances would probably improve the kernel estimation as well as model extrapolations.

Another potential limitation of the experiment is related with the aerobiology of *M. fijiensis* spores. In theory, two major differences between experimental and natural plantation conditions may have had an aerological influence on spore release and transport. The first one is related with the phenotype (e.g., height and shape) of the sources of dispersal. Our artificial banana plants (ascospore source of dispersal) were on that sense pretty well representative of natural banana trees which should limit such potential bias. The second one is linked to the plant density as it directly influences local air movements [89]. On the ascospore experimental site, we concentrated the amount of inoculum (i.e., necrotic tissue) of ≈50 natural plants on 5 artificial trunks to limit at maximum the spore emission area surface. In addition, the density of plants over the entire study site was much lower than what can be observed in natural banana plantations. It is thus possible that such a lower plant density in our experimental site, as compared with natural conditions, may influence the dispersal kernel estimation. Further investigation of spore dispersal patterns through mechanistic models by wind would be needed to specifically assess this question.

### Direct *vs* indirect estimation of dispersal

Direct estimations of dispersal kernel have previously been criticized for two main reasons. First, when they rely on physical traps, they inform on the dispersal of particles independently on their viability. Here, because we used biological traps, our kernel reflects “effective dispersal”, including release, transport, deposition and establishment on a leaf. Second, direct measurements can be sensitive to the ecological and environmental conditions of the location and period of the experiment. Dispersal kernels are thus expected to be valid under the experimental conditions but evaluating their generality for the species is challenging [1]. One way to investigate the repeatability of our results would have been to perform the experiment a second time. This was unfortunately not feasible in our case because of the consequent contamination of the banana plant traps by the source of inoculum and also for logistic reasons. An alternative way to gain insights on the validity of our estimation can potentially be achieved by comparing the kernel estimated with an indirect measure of gene flow which integrates migration movements over several generations. From our knowledge, this has never been realized for a plant pathogen species before (see Table S11 in File S1 for a few examples found in few other species). The indirect estimate of the parent-offspring dispersal distances obtained in Rieux et al. [67] is ≈6-fold more important (*σ* = 1.2 km/generation^1/2^) than the demographic estimate computed over the 8 directions from the dispersal kernel (*σ* = 201 m/generation^1/2^). This ratio appears consistent with the values measured in other species (Table S11 in File S1). Nevertheless, several factors can explain discrepancies between our direct and indirect estimations. First, environmental conditions could differ if experiments are realized in contrasted location/season. This should not be a major issue here as the indirect estimation has been realized in a neighbouring area (i.e., 50 km away from the direct experimental site) and obtained by averaging dispersal processes over ≈2 years, thus integrating a wide range of environmental conditions. Second, most classical indirect approaches are expected to be sensitive to leptokurtic dispersal [68], which might explain differences between direct and indirect estimates in case of fat-tailed dispersal kernel. This is neither supposed to hold here because Rieux et al. [67] used simulations to show that their neutral genetic approach was robust to deviation from a Gaussian kernel, even in the case of extremely high kurtosis. Third, the difference in number of generations during which the estimation is realized can also be a source of disparity. Because the kernel was measured over a single generation, one may want to convolve (i.e., reproduce) it 15 times to properly compare the two values. Such a procedure is not trivial and has not been implemented here but previous theoretical results allow predicting qualitatively the pattern we would obtain. It has indeed been shown that fat-tailed kernel (as measured in the present study) generate increasing speeds during colonization [2]. This suggests that the average distance travelled and the standard deviation of axial dispersal distances should be higher if computed from a 15 times convolved kernel in comparison to single generation estimation. Last, the indirect estimation could also have been over-estimated if integrating potential human mediated transport of infected material [67]. This is likely to occur in this area where plant exchanges occur commonly between villages separated by several kilometres. Altogether those arguments suggest that accounting for the above listed factors may lead to a reduction of the ratio between *M.fijiensis* direct and indirect estimates of dispersal, which constitutes a good case for them to be biologically relevant.

### New insights into BLSD management

The results we obtained improve our understanding of *M.fijiensis* epidemiology. The observed combination of short and long distance spore dispersal has significant consequences in terms of disease spread. Indeed, long distance ascospore dispersal is expected to generate new populations far (at least 1 km) from the front. A subsequent rapid growth via conidia may contribute to the rapid establishment of such populations at the edge of the expansion front because this pathogen is heterothallic and needs high population densities for sexual reproduction. The iteration of both processes, known as “stratified dispersal combination” is expected to generate a mosaic of relatively homogeneous genetic patches and to accelerate BLSD spatial expansion [4], [90], [91]. Interestingly, Rieux et al. [92] reported such a patchy genetic structure in a neighbouring area. A cautious use of the kernels estimated in the current study (e.g., truncating the ascospore kernel tail over distances >>1 km) is likely to be helpful for the design of future BLSD management policies. For instance, it could help to better predict the patterns of disease propagation at the local parcel scale. Also, the major influence of ascospore *vs* the local contribution of conidia underlined in the current study support currently applied management guidelines in commercial banana plantations, which consist in thinning out necrotic leaves from which ascospores are produced [93] to slow down the diffusion of the disease. Our results might thus be helpful to make current fungicide resistance management strategies more sophisticated [26], [59]. Indeed, dispersal processes influence the diffusion of both sensitive and resistant strains between parcels and consequently the evolutionary potential of pathogen populations. To illustrate this point, Lernormand & Raymond [24] theoretically demonstrated that management strategies based on gene flow between untreated and treated areas are potentially efficient to prevent resistance genes frequency reaching high equilibrium value in treated areas. In West-African agricultural landscapes, the important dispersal abilities of ascospores may induce asymmetric gene flow from surrounding untreated food-crop plantations, where ascospore population size is very large, towards commercial plantations where only conidia are found as a consequence of common cultural practices [94]. This may represent a favourable situation for setting up an integrated management of fungicide resistances relaying on refuge strategy, through a rational management of treated and untreated areas. For that purpose, integrating the estimated kernel of ascospores and conidia in theoretical and spatially explicit models (e.g., including both the sizes and position of treated and untreated parcels at a landscape scale) could help predicting spatial patterns of fungicide resistance evolution under different management strategies.

### Conclusions

Our study on the *Mycosphaerella fijiensis*/banana plant patho-system illustrates the benefits of combining several technical and statistical precautions in the design of experiment and data analysis to get a direct estimation of wind-dispersed fungal pathogens dispersal kernel, including some of its long-distance dispersal component. Obtaining such landscape-scale estimates of contemporary dispersal in a wider range of species and environments is crucial for ecological and evolutionary principles to guide the design of quarantine and management policies of invasive crop pests. In the specific case of *M.fijiensis*, a cautious use of the kernels estimated should be helpful to support local landscape scale strategies aiming to manage fungicide resistance or the deployment of new banana plant resistant varieties.

## Supporting Information

File S1 **Supporting files.** **Appendix S1,** Increasing traps receptive surface with distance on the ascospore experimental site. **Appendix S2,** Details on the production of resistant inoculum sources. **Appendix S3,** Details on the intensity of inoculum sources estimation. **Appendix S4,** Details on the molecular assessment of QoI resistance. **Appendix S5,** Details on the dispersal model likelihood calculation. **Appendix S6,** Details on the modelling of anisotropy in density and distance. **Appendix S7,** Comparison between observed and predicted density of resistant lesions across the 8 cardinal directions on the ascospore site. **Appendix S8,** Details on the comparison with an indirect (genetic-based) estimate of *M. fijiensis* dispersal in Cameroon. **Appendix S9,** Wind record patterns. **Appendix S10,** Correlation between wind patterns, distance travelled & density of spores. **Appendix S11,** Confrontation between demographic and genetic estimates found in the literature.
(DOCX)

File S2 **R code used for statistical analyses.**
(TXT)

File S3 **Data set for both ascospores and conidia experimental sites.**
(RAR)
