# Supplementary material for: Long-Distance Wind-Dispersal of Spores in a Fungal Plant Pathogen: Estimation of Anisotropic Dispersal Kernels from an Extensive Field Experiment
Source: PLoS One. 2014 Aug 12;9(8):e103225. doi: 10.1371/journal.pone.0103225 (PMC4130500; doi:10.1371/journal.pone.0103225)
Supplement: File S1 — Supporting files. Appendix S1, Increasing traps receptive surface with distance on the ascospore experimental site. Appendix S2, Details on the production of resistant inoculum sources. Appendix S3, Details on the intensity of inoculum sources estimation. Appendix S4, Details on the molecular assessment of QoI resistance. Appendix S5, Details on the dispersal model likelihood calculation. Appendix S6, Details on the modelling of anisotropy in density and distance. Appendix S7, Comparison between observed and predicted density of resistant lesions across the 8 cardinal directions on the ascospore site. Appendix S8, Details on the comparison with an indirect (genetic-based) estimate of M. fijiensis dispersal in Cameroon. Appendix S9, Wind record patterns. Appendix S10, Correlation between wind patterns, distance travelled & density of spores. Appendix S11, Confrontation between demographic and genetic estimates found in the literature. (DOCX) [file pone.0103225.s001.docx]

**File S1 Contents:**

2

3

5

8

11

13

15

16

19

20

21

22

**Appendix S1.** Increasing traps receptive surface with distance on the ascospore experimental site……………………………………………………………………………………………………………………

**Appendix S2.** Details on the production of resistant inoculum sources…………………………………

**Appendix S3.** Details on the intensity of inoculum sources estimation…………………………..……...

**Appendix S4.** Details on the molecular assessment of QoI resistance……………………………………

**Appendix S5.** Likelihood calculation of the dispersal model…………………………………………………..

**Appendix S6.** Details on the modelling of anisotropy in density and distance………………………..

**Appendix S7.** Comparison between observed and predicted density of resistant lesions across the 8 cardinal directions………………………………………………………………………………………………

**Appendix S8.** Details on the comparison with an indirect (genetic-based) estimate of *M. fijiensis* dispersal in Cameroon……………………………………………………………………………………………….

**Appendix S9.** Wind record patterns……………………………………………………………………………………….

**Appendix S10.** Correlation between wind patterns, distance travelled & density of spores……

**Appendix S11.** Confrontation between demographic and genetic estimates found in the literature………………………………………………………………………………………………………………………………..

**References cited**…………………………………………………………………………………………………………………….

**Appendix S1. Increasing traps receptive surface with distance on the ascospore experimental site.**


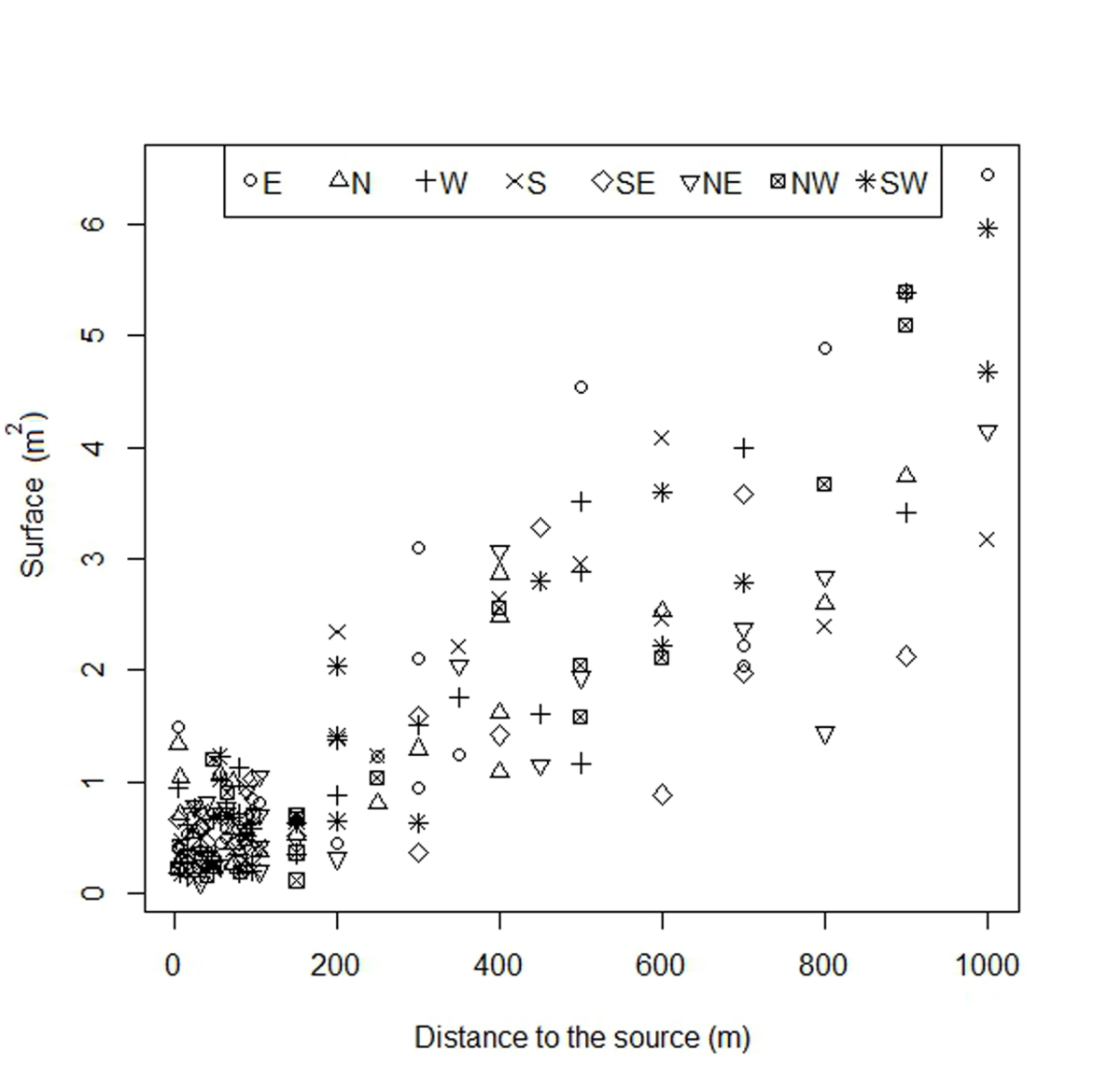


**Figure S1**. Increasing traps receptive surface with distance on the ascospore experimental site.

**Appendix S2. Details on the production of resistant inoculum sources.**

To obtain an inoculum source highly resistant to QoI fungicides, 50 banana plants were sprayed weekly, from January to September 2010, with Bankit 25 SC at the rate of 100 g azoxystrobin/ha in 20 l of water and using 1‰ Triton X 100 as a wetting agent. These banana plants were located more than 50 km away from the experimental site to avoid contamination from this source. The frequency of resistant strains increased from 0% in January 2010 to 96% and 98% the day before starting the experiment for lesions bearing conidia and ascospores, respectively.


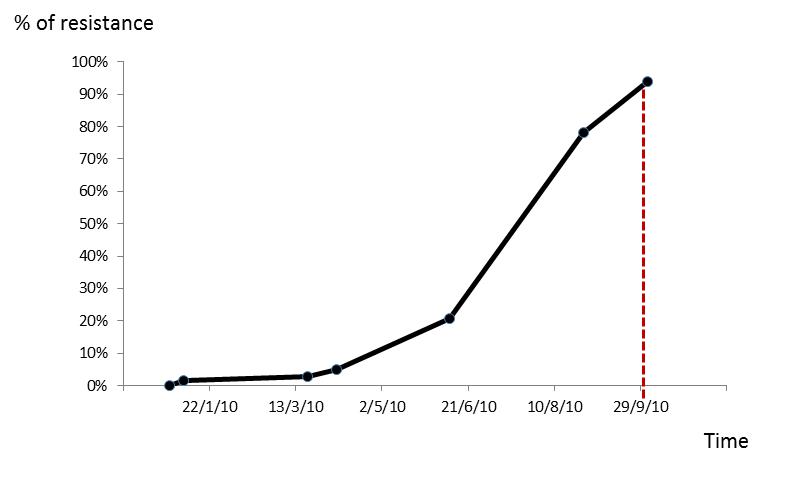


**Figure S2-1**. Evolution of the QoI- resistant allele frequency in the banana plot where the source of inoculum has been produced. Fungicide treatments with azoxystrobin were realized once a week during 9 months at a frequency of 1spray/week (see text for details). The red line indicates time at which source has been prepared.

The two different sources of inoculum were subsequently prepared from those high-frequency fungicide-resistant strains samples. For ascospores, leaves carrying necrotic tissue in which ascospores are produced and not conidia were cut from 50 sprayed banana plants. Necrotic tissue was cut from the leaves and stapled to artificial leaves made of steel and mosquito net (See pictures in figure S2-2). We installed 48 artificial leaves (80x40 cm) on 5 plastic trunks. This provided a large surface of necrotic tissue (12 m^2^ in total) over a small soil area (16 m^2^). For conidia, we selected 9 banana plants on which all necrotic tissue had been removed. The remaining leaves bore lesions exclusively of young stages (stage 1 to 3) which prevent the formation of ascospores during the experiment. These 5 banana plants were uprooted and transplanted to the experimental plot.


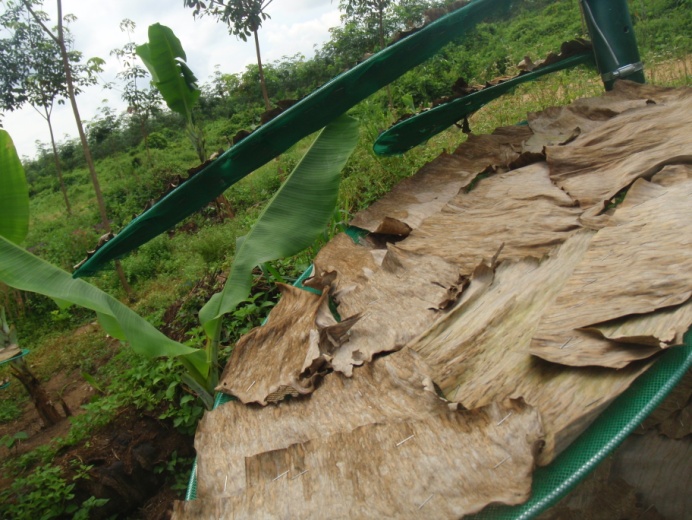

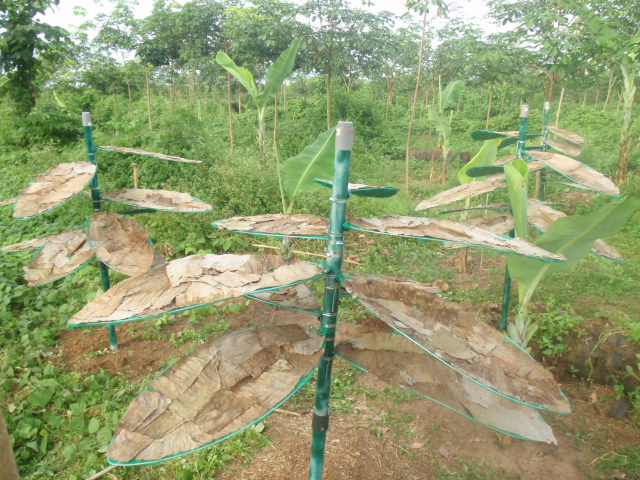

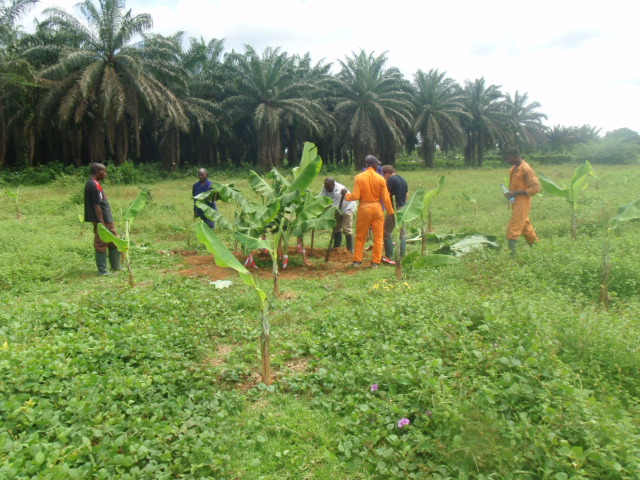

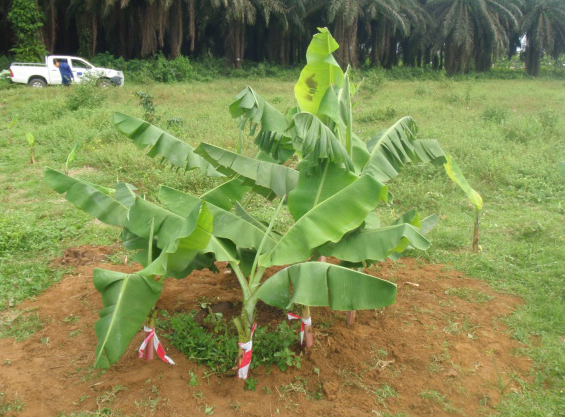


**Figure S2-2.** Pictures of the resistant sources of inoculums used on the two experimental plots. The top panel pictures represent the artificial source of ascospores and the bottom panel pictures illustrate the natural source of conidia.

**Appendix S3. Details on the intensity of inoculum sources estimation.**

The potential intensity of the sources has been estimated both experimentally and statistically (i.e, jointly with the dispersal kernel inference). Below, we provide details on the experimental procedure and further compare values obtained between the two methods.

Experimental measurement

*Method*

Four days before the installation of the inoculum sources, leaf samples (either necrotic tissues or limb pieces bearing young lesions) were collected on 10 of the 50 banana plants sprayed with azoxystrobin. We experimentally estimated the average number of spores potentially released by the two types of inoculum sources through meticulous lab counting as follow:

**Source of ascospores.** On each banana plant, two necrotic tissues of 2.2 cm in diameter were cut with a pastry cutter and stapled to a filter paper disc 90 mm in diameter. The filter papers were immersed in distilled water for 5 min and then placed in the lid of a Petri dish agar plate for two hours to induce ascospore discharge. After this step, the filter papers were removed and, after 48 h, the number of ascospores per disc was counted under a microscope. The average value of this number was taken as an estimate of the number of ascospores produced per surface of necrotic area in the source.

**Source of conidia.** On each banana plant, 1 leaf disc of 2.2 cm in diameter was cut with a pastry cutter in a place where young lesions were sufficiently dense (about 5 lesions/cm^2^), and the number of lesions/disc was counted. From lesion counting we estimated that each banana tree of the source bore 1,000 lesions at stage 2. In order to remove all conidia from the lesions, discs were applied successively 6 times over a Petri dish agar plate. The number of conidia/disc was counted 48 h later under a microscope. The number of conidia produced per lesion was then calculated from this value, and the number of conidia produced by the 5 banana plants of the source was then subsequently estimated.

*Results*

Experimentally, we observed a mean value of 241 ascospores produced per leaf disc, and 36 conidia per lesion. This resulted in a potential experimental source intensity of 7x10^6^ ascospores and 1.8x10^5^ conidia.

Model estimation

As detailed in the statistical paragraph of the material and methods section, the statistical model considered to fit theoretical dispersal kernels to disease gradients allowed us to estimate *s_0_*, defined as the number of spores released outside of the source area (namely the source intensity). We estimated *s_0_* = 2.3x10^7^ CI [2.02x10^7^ – 4.05x10^7^] and *s_0_* = 1.96x10^4^ CI [2.9x10^3^ – 1.05x10^5^] for the ascospores and conidia source intensities, respectively.

Comparison between experimentally- and statistically-estimated source intensities

Contrasted ratio between experimental and model-based estimation of source intensity were thus observed between the two types of spores.

For ascospores, both estimations indicate that a high number of propagules were released (*i.e.*, 7 and 23 millions for the lab and model-based estimation, respectively). A smaller value given by the lab measurement was expected because it provides an instantaneous estimate of the number of ascospores released during one single event of ascospore realease, whereas model estimates reflect the accumulated number of ascospores released throughout the period during which the inoculum was in place. The source was removed 17 days after the beginning of the experiment and several rainfall events occurred during this period. Since ascospore release is induced by rain (Gauhl, 1994) it is then probable that several ascospore discharges occurred during this period, which could explain the difference between the two estimates. Burt (2003) proposed a relationship to estimate the number of ascospores released from necrotic area measurement. According to their method, the intensity of the source would have been greater (64 million of ascospores), probably because their estimation account for ascospores released until exhaustion of necrotic tissues.

For conidia, the number of conidia released by the source estimated from the model was much smaller than the one measured in the laboratory. This indicates that the majority of conidia did not disperse out of the central source plot. Rain splash may have limited the dispersion of conidia (Gauhl 1994) at the source scale (*i.e.*, at distances < 2.5m). Another plausible explanation could be that many conidia were not dispersed because they could not be dislodged due to insufficient wind forces

**Appendix S4. Details on the molecular assessment of QoI resistance.**

QoI (belonging to the well-known family of strobilurins) are systemic fungicides that inhibit mitochondrial respiration of fungi by binding to the cytochrome bc1 enzyme complex (complex III) at the Qo site (Bartlett et al., 2002). We detail below the approach considered to detect and assess the frequency of resistance in *M. fijiensis* samples.

Samples preparation and DNA extraction

For each sampled lesion, a standardized fragment (2 mm in diameter) was cut in the laboratory and dried for 1 day at 55°C before being stored at –20°C. A maximum of 10 lesion fragments from the same plant was bulked for DNA extraction according to the procedure detailed in Robert et al. (2010).

PCR

In *M. fijiensis*, QoI resistance is conferred by a single mutation in the mitochondrial cytochrome *b* (cyt *b*) gene (Sierotzki et al., 2000). This mutation leads to an amino acid change from glycine to alanine at codon 143 (G143A). A 77-bp DNA fragment including the G143A mutation was amplified by PCR using the following primer sequences (designed on the basis of NCBI Accession N° AF343070):

Primer forward: 5’ TGGGTATGTTTTACCTTATGGTC 3’

Primer reverse : 5’ biotin_AGGGTATAGCGCTCATT 3’

Fragment amplified: including the G143A SNP


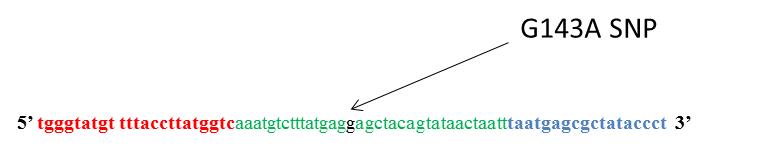


PCR was performed using the HotStarTaq Master mix kit (Quiagen) under the following program:

Step Temperature Time

1. 94°C 15‘ (min)

2. 94°C 30‘‘ (sec)

3. 55°C 30‘‘(sec)

4. 72°C 1‘(min)

5. Goto 2 35 times

6. 72°C 10‘ (min)

7. end

G143A mutation point frequency assessment through pyrosequencing

The mutation point frequency for each sample of *M. fijiensis* was assessed using a pyrosequencing technique at Bayer Crop Science Laboratory.

Pyrosequencing primer (sequencing primer, forward)

MF03_for_seq 5’ GTCAAATGTCTTTATGAG 3’

Within such pyrosequencing assays, dNTPs are added sequently to the prepared single stranded fungal DNA. Each incorporation causes a light flash at the end of an enzyme cascade. Specific software calculates the allele frequency at the position of the possible mutation in %, thus indicating the grade of resistance towards QoI fungicides.

An enzyme-cascade system, consisting of four enzymes, DNA polymerase, ATP sulfurylase, luciferase and apyrase and specific substrates, adenosine 5´ phosphosulfate (APS) and luciferin, produces light whenever a nucleotide forms a base pair with the complementary base in a DNA template strand. The light signal is detected and seen as a peak in a Pyrogram. The height of each peak is proportional to the number of nucleotides incorporated. After injection of one out of four dNTPs the DNA polymerase catalyses the extension of the sequencing primer complementary to the single stranded DNA template. As a result of one single dNTP incorporation, one pyrophosphate (PPi) is produced. ATP sulfurylase catalyses the chemical reaction of adenosine 5´ phosphosulfate and pyrophosphate to ATP. This ATP activates the luciferase induced conversion of luciferin to oxyluciferin resulting in the release of a light flash. The amount of light produced is proportional to the number of incorporated nucleotides. The enzyme apyrase degrades continuously all unincorporated dNTPs in parallel to the former described reactions. When adding the next dNTP, all dNTPs of the previous step were completely degraded.


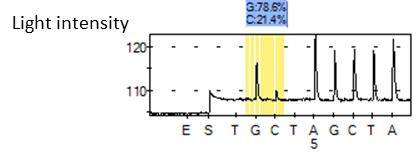


**Figure S4.1** Example Pyrogram, sample containing 78.6% wild (sensitive)-type copies

From such a pyrogram, the ratio of mutated (G143A, resistant) to wild type (sensitive) DNA within each sample can be determined.

**Appendix S5. Likelihood calculation of the dispersal model**

The total number of resistant lesions (TNRL) on a leaf located at (*x*, *y*) follows either a Poisson distribution with mean *S*(*x*, *y*), the infectious potential at location (*x*, *y*) (see Eqn. (1) in the main text), or a negative-binomial distribution with mean *S*(*x*, *y*) and dispersion parameter $\tau$ (in this case, the variance of TNRL is $S(x, y)+ {S(x, y)}^{2}/\tau$).

Under the Poisson assumption, the probability distribution function of TNRL is, for *n* in {0,1,2,…}:

$p_{\mathrm{TNRL}}\left( n|x,y \right)=\exp\left( -S(x, y) \right)\frac{{S(x, y)}^{n}}{n!}.$ Eqn S5.1

Under the negative-binomial assumption, the probability distribution function of TNRL is, for *n* in {0,1,2,…}:

$p_{\mathrm{TNRL}}\left( n|x,y \right)=\frac{\Gamma(n+\tau)}{\Gamma(\tau)n!}\left( \frac{\tau}{\tau+S(x, y)} \right)^{\tau}\left( 1-\frac{\tau}{\tau+S(x, y)} \right)^{n},$ Eqn S5.2

where $\Gamma$ is the gamma function.

The number of genotyped resistant lesions (GNRL) on a leaf located at *(x,y)* follows a hypergeometric distribution whose parameters are (i) the population size, equal to the total number of lesions sampled on this leaf (TNL), (ii) the number of success states in the population, equal to the total number of resistant lesions (TNRL), and (iii) the number of draws (without replacement), equal to the number of genotyped lesions (GNL). The probability distribution function of GNRL given TNL, TNRL and GNL is, for *k* in {0,1,...,GNL}:

$q_{\mathrm{GNRL}|TNL,TNRL,GNL}\left( k|TNL,TNRL,GNL,x,y \right)=\frac{\left( \begin{aligned} \mathrm{TNRL} \\ k \end{aligned} \right)\left( \begin{aligned} TNL-TNRL \\ \mathrm{GNL}-k \end{aligned} \right)}{\left( \begin{aligned} \mathrm{TNL} \\ \mathrm{GNL} \end{aligned} \right)},$ Eqn S5.3

where $\left( \begin{aligned} a \\ b \end{aligned} \right)$ is a binomial coefficient.

This leads to the probability distribution function of GNRL given TNL and GNL that is equal to, for *k* in {0,1,...,GNL}:

$$\begin{matrix} q_{\mathrm{GNRL}|TNL,GNL}\left( k|TNL,GNL,x,y \right)=\sum_{n=0}^{\infty} q_{\mathrm{GNRL}|TNL,TNRL,GNL}\left( k|TNL,n,GNL,x,y \right)p_{\mathrm{TNRL},x,y}\left( n,x,y \right) \\ =\sum_{n=0}^{\mathrm{TNL}} q_{\mathrm{GNRL}|TNL,TNRL,GNL}\left( k|TNL,n,GNL,x,y \right)p_{\mathrm{TNRL},x,y}\left( n,x,y \right). \end{matrix}$$

Eqn S5.4

The summation is reduced to integers from 0 to TNL since TNRL is constrained to be less or equal than TNL.

Let (*x_i_,y_i_*), TNL*_i_*, GNL*_i_* and GNRL*_i_* denote data corresponding to sampled leaf *i* in {1,…,I}. The likelihood of the model is:

$\prod_{i=1}^{I} q_{\mathrm{GNRL}|TNL,GNL}\left( \mathrm{GNRL}_{i}|\mathrm{TNL}_{i},\mathrm{GNL}_{i},x_{i},y_{i} \right).$ Eqn S5.5

**Table S5** AIC values obtained assuming both the Poisson and the Negative-binomial distributions for the different dispersal kernels tested. Lowest AIC (best models) are highlighted in bold.

**Appendix S6. Details on the modelling of anisotropy in density and distance.**

Dispersal is anisotropic if the behaviour of the dispersed propagules depends on the direction. This angular dependence can occur in various ways. Here, we focus on two sorts of dependence represented by two independent summary functions characterizing dispersal in density and distance, respectively (Soubeyrand et al. 2007). The first describes the direction taken by the spores during transit, whereas the second provides the expected distance between the spore’s source and the location of its deposition, given the direction taken by the spore. We used von Mises function (Fisher 1995) to model those two anisotropy functions. Note that von Mises function is a unimodal function (*i.e.*, there is only one main dispersal direction) that is not constrained by any prerequisite model.

Modeling anisotropy in density

Let *f* denote the von Mises directional density function, reflecting the probability that a spore is deposited in an angle sector *dϕ* radiating from the source in direction ϕ ∈ [0-2π[ (See Soubeyrand et al. 2007 for details):

Eqn S6.1

Eqn S6.2

Parameter *μ* is the expected dispersal direction, and *δ* represents the variability of dispersal directions around*μ*. For *δ* = 0 dispersal directions are distributed uniformly in [0-2π[ , and for *δ* → +∞ dispersal directions are all equal to *μ*. *I*_0_ is an integrative constant.

Modeling anisotropy in distance

Function *g* denotes the expected distance function and is chosen proportional to a Von Mises function.

Eqn S6.3

where *v* is the direction with largest mean dispersal distance and *k* controls the variability of the expected dispersal distance as direction varies. For *k*=0 dispersal distances are the same whatever the dispersal direction. *g*_0_ is a multiplicative constant measuring how far from the source spores are deposited.

Computing the mean distance travelled by spores

Under the dispersal kernel *K*, the mean distance travelled can be obtained assuming that dispersal is lower than or equal to $\rho$^max^>0 and is equal to

Eqn S6.4

In our computations, we used two different value of $\rho$^max^. First we considered $\rho$^max^=1000 (1000m being the distance between the source and the most distant site) and $\rho$^max^= Infinite (no limitation)

**Appendix S7. Comparison between observed and predicted density of resistant lesions across the 8 cardinal directions.**

**Figure S7.** The graph in the middle illustrates the observed density in resistant lesions as a function of the distance from the source of the ascospore experimental site (c.f. Fig 2.B in main document). One supplemental graph has been built for each of the 8 directions in which the predicted values for the density in resistant lesions predicted by the exponential power kernel (red line, see parameter in Table 1) are compared to the values observed in the field (black dots).

**Appendix S8. Details on the comparison with an indirect (genetic-based) estimate of *M. fijiensis* dispersal in Cameroon**

Details on the indirect estimation (Rieux et al. 2013)

Rieux et al. (2013), proposed a method to infer contemporary dispersal from the temporal variation in neutral markers cline shape in a contact zone. They applied to the case of *Mycosphaerella fijiensis* in which a secondary contact zone was clearly detected using 15 SSRs loci in South-West Cameroon. The sampling area considered in Rieux et al. (2013) and the experimental site of the present study are relatively close (distance ~ 50 Km). The estimation was realized using two temporal samplings, the first one being realized in November 2005, the second one in February 2007. Such a time interval has been estimated to be equivalent to 15 sexual generations of the pathogen. Using neutral genetic clines decay in 2D, they find that *σ*, the standard deviation of parent-offspring dispersal distances along the main cline axis is equals to 1.2 km/generation^1/2^, support limit interval [0.7km-1.6km]. They further used simulation to show that their estimation procedure, which assumes diffusion approximation, is robust to dispersal kernel with high kurtoses.

Comparison between direct and indirect estimate of dispersal

The mean dispersal distances calculated from the kernel and from equation 6 are equivalent to the mean of **euclidian** distances travelled by the spores whereas *σ* is defined as the standard deviation of **axial** dispersal distances. Aiming to compare both quantities appears thus meaningless. A better strategy would be to compute *σ* from the estimated kernel.

Computation of *σ* from the dispersal kernel

The computation of *σ* from a 2D anisotropic kernel is not as straightforward as for the average dispersal distance and requires careful computations as described below. First, *σ* is by definition a variable calculated in 1D (projected on 1 axis). Assuming an isotropic kernel, if X_1_ represent the x-axis, the expected value of axial dispersal distance E(X_1_) is zero and one may compute *σ* projected on the x-axis. However, in our case of anisotropic kernel, computing *σ* on X_1_ is meaningless. One possibility to compare dispersal measured between direct and indirect method would be to compute an average value *σ* from the eight values calculated on each of axis radiating from the source on the ascospore experimental site.

We define *σ*^2^(*ϕ*_0_) as the variance of the coordinate x(*ϕ*_0_) along the axis with angle *ϕ*_0_ with respect to the abscissa axis. Thus, *σ*^2^(*ϕ*_0_) varies with the angle *ϕ*_0_. The coordinate x(*ϕ*_0_) along the axis with angle *ϕ*_0_ is equal to *ρ* cos(*ϕ*-*ϕ*_0_), where (*ρ,ϕ*) are the polar coordinates of the location of an offspring spore. Therefore, the variance of dispersal *σ*^2^(*ϕ*_0_) satisfies:

Eqn S8.1

In our study we aim to compute the conditional variance of dispersal *σ*^2^(*ϕ*_0_ | *ρ*≤*ρ*_max_) given that the dispersal is lower than or equal to a given distance *ρ*_max_>0:

Eqn S8.2

Where:

Eqn S8.3

And:

Eqn S8.4

and where *K* is the dispersal kernel. These terms can be easily computed with standard numerical integration techniques. We used $\rho$_max_ = 1000 (1000m being the distance between the source and the most distant site). As directly performed on primary disease gradient data observed over a single pathogen life cycle, the estimated rate of dispersal can be expressed in “per-generation rate”.

Values of *σ* computed from the dispersal kernel

**Table S8** Values of *σ* and 95% confidence interval computed from the dispersal kernel using equation Eqn S6.2. Values are given in m/generation^1/2^.

**Appendix S9. Wind record patterns.**

**
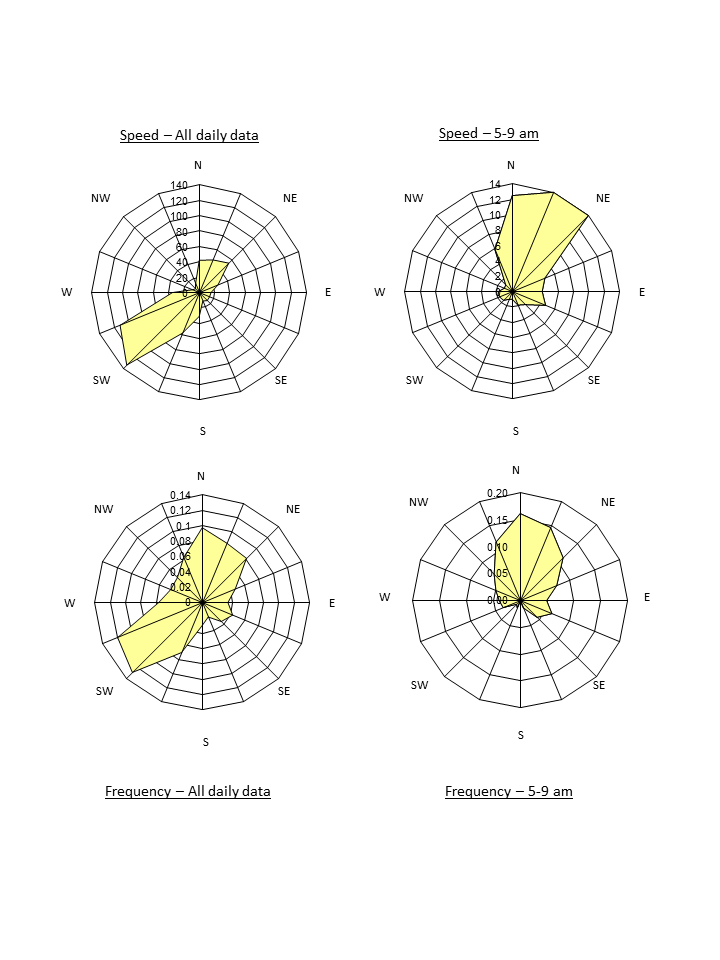
**

**Figure S9.** Cumulative wind speed and frequency of wind record for each direction between D_0_ (5/10/2010) and D_17_ (19/10/2010). Both indices were either calculated considering all daily data or considering only data recorded between 5 and 9 a.m.

**Appendix S10. Correlation between wind patterns, distance travelled & density of spores**

|  |  |  |
| --- | --- | --- |
| Ascospores | distance travelled | density |
| Wind frequency (total) | 0.06 (NS) | 0 (NS) |
| Wind speed (total) | 0.36 (NS) | 0.02 (NS) |
| Wind frequency (5-9 a.m) | 0.50 (NS) | 0.50 (NS) |
| Wind speed (5-9 a.m) | 0.65 (NS) | 0.57 (NS) |
|  |  |  |
| Conidia | distance travelled | density |
| Wind frequency (total) | **0.91 (***)** | **0.95 (***)** |
| Wind speed (total) | 0.69 (NS) | **0.70 (*)** |
| Wind frequency (5-9 a.m) | 0.29(NS) | 0.33 (NS) |
| Wind speed (5-9 a.m) | 0.09 (NS) | 0.28 (NS) |
|  |  |  |

**Table S10.** Spearman rank correlation coefficient (and the associated two-sided *p*-value, NS >0.05,* P<0.05; *** P<0.0001) between spores density or distances travelled and wind patterns in a given direction. Significant values are highlighted in bold.

**Appendix S11. Confrontation between demographic and genetic estimates found in the literature**

**Table S11.** Confrontation between demographic and genetic estimates found in the literature

**References cited:**

Bartlett D., Clough J.M., Godwin J., Hall A., Hamer M. & Parr-Dobrzanski B. (2002). The strobilurin fungicides. *Pest Management Science*, 58, 649-662.

Fisher N.I. (1995). *Statistical Analysis of Circular Data*.

Gauhl F. (1994). *Epidemilogy and ecology of black sigatoka* INIBAP edn. INIBAP, Montpellier.

Burt PJA (2003) Airborne dispersal of Mycosphaerella fijiensis. In: Mycosphaerella Leaf Spot Diseases of Bananas: Present Status and Outlook. Proceedings of the Workshop on Mycosphaerella Leaf Spot Diseases, San José, Costa Rica.

Lachish S., Miller K.J., Storfer A., Goldizen A.W. & Jones M.E. (2011). Evidence that disease-induced population decline changes genetic structure and alters dispersal patterns in the Tasmanian devil. *Heredity*, 106, 172-182.

Lenormand T., Guillemaud T., Bourguet D. & Raymond M. (1998). Evaluating gene flow using selected markers: A case study. *Genetics*, 149, 1383-1392.

Mallet J., Barton N., Lamas G., Santisteban J., Muedas M. & Eeley H. (1990). Estimates of selection and gene flow from measures of cline width and linkage disequilibrium in heliconius hybrid zones. *Genetics*, 124, 921-936.

Oddou-Muratorio S. & Klein E.K. (2008). Comparing direct vs. indirect estimates of gene flow within a population of a scattered tree species. *Mol. Ecol.*, 17, 2743-2754.

Pepperell C.S., Granka J.M., Alexander D.C., Behr M.A., Chui L., Gordon J., Guthrie J.L., Jamieson F.B., Langlois-Klassen D., Long R., Nguyen D., Wobeser W. & Feldman M.W. (2011).

Dispersal of Mycobacterium tuberculosis via the Canadian fur trade. *Proceedings of the National Academy of Sciences of the United States of America*, 108, 6526-6531.

Rieux A., Lenormand T., Carlier J., De Lapeyre De Bellaire L. & Ravigne V. (2013). Using neutral cline decay to estimate contemporary dispersal: a generic tool and its application to a major crop pathogen. *Ecology Letters*.

Robert S., Rieux A., Argout X., Carlier J. & Zapater M.F. (2010). Optimized genotyping with microsatellite markers in the fungal banana pathogen *Mycosphaerella Fijiensis*. *American Journal of Botany*, 97.

Rousset F. (2000). Genetic differentiation between individuals. *Journal of Evolutionary Biology*, 13, 58-62.

Sierotzki H., Parisi S., Steinfeld U., Tenzer I., Poirey S. & Gisi U. (2000). Mode of resistance to respiration inhibitors at the cytochrome bc(1) enzyme complex of *Mycosphaerella fijiensis* field isolates. *Pest Management Science*, 56, 833-841.

Soubeyrand S., Enjalbert J., Sanchez A. & Sache I. (2007). Anisotropy, in density and in distance, of the dispersal of yellow rust of wheat: Experiments in large field plots and estimation. *Phytopathology*, 97, 1315-1324.

Sumner J., Rousset F., Estoup A. & Moritz C. (2001). 'Neighbourhood' size, dispersal and density estimates in the prickly forest skink (*Gnypetoscincus queenslandiae*) using individual genetic and demographic methods. *Mol. Ecol.*, 10, 1917-1927.
